# Lipoprotein(a) and the atherosclerotic burden – Should we wait for clinical trial evidence before taking action?

**DOI:** 10.1016/j.athplu.2024.09.004

**Published:** 2024-09-26

**Authors:** Isabella Fichtner, Chiara Macchi, Alessandra Stefania Rizzuto, Stefano Carugo, Alberto Corsini, Massimiliano Ruscica

**Affiliations:** a Department of Pharmacological and Biomolecular Sciences "Rodolfo Paoletti", Università Degli Studi Di Milano, Milan, Italy; b Department of Clinical Sciences and Community Health, Università Degli Studi Di Milano, Milan, Italy; c Department of Cardio-Thoracic-Vascular Diseases, Foundation IRCCS Ca' Granda Ospedale Maggiore Policlinico, Milan, Italy

**Keywords:** Lipoprotein(a), Atherosclerotic cardiovascular risk, lipoprotein(a) measurements

## Abstract

The fact that lipoprotein(a) levels should be regarded as a causal residual risk factor in the atherosclerotic cardiovascular diseases (ASCVD) is now a no-brainer. This review article aims to summarize the latest evidence supporting the causal role of lipoprotein(a) in ASCVD and the potential strategies to reduce the lipoprotein(a) burden until clinical trial results are available. Epidemiological and genetic data demonstrate the causal link between lipoprotein(a) and increased ASCVD risk. That being said, a specific question comes to mind: “must we wait for outcome trials in order to take action?”. Given that lipoprotein(a) levels predict incident ASCVD in both primary and secondary prevention contexts, with a linear risk gradient across its distribution, measuring lipoprotein(a) can unequivocally help identify patients who may later benefit from specific lipoprotein(a)-lowering therapies. This understanding has led various National Societies to recommend dosing lipoprotein(a) in high-risk individuals and to support the recommendation of measuring lipoprotein(a) levels at least once in every adult for risk stratification.

## Highlights

• Lipoprotein(a) is a significant contributor in the residual risk of ASCVD
• Lipoprotein(a) should be measured in all adults at least once in life
• About one-third of FH patients have elevated levels of lipoprotein(a)
• Efforts are needed to overcome the common barriers to ordering lipoprotein(a) test

## Graphical abstract

**Figure:**
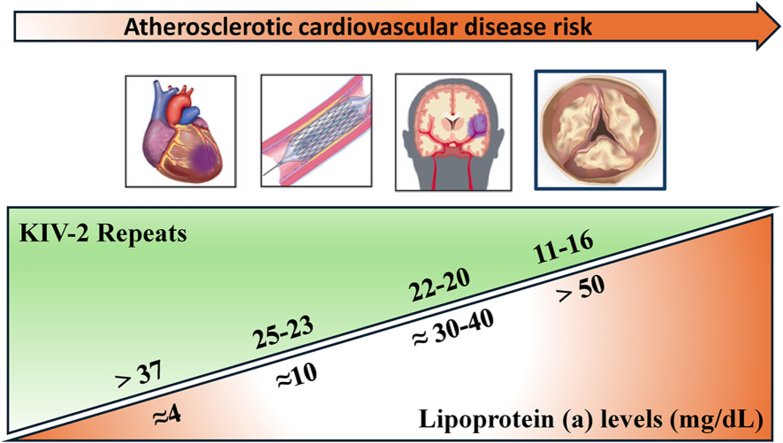

## 1 Introduction

In recent years, lipoprotein(a), the most polymorphic of all lipoproteins [1], has been recognized as a significant contributor in the residual risk of atherosclerotic cardiovascular diseases (ASCVD) [2]. Epidemiological and genetic studies have raised awareness of the association between ASCVD risk and hyperlipoproteinaemia(a) [3]. This has shed light on the need for improvements in treatments for patients with ASCVD, as current therapies are incapable of lowering lipoprotein(a) to beneficial levels from a cardiovascular standpoint [4]. Despite lifestyle interventions and pharmacological strategies reducing low-density lipoprotein cholesterol (LDLc), ASCVD events remain frequent, even among individuals with controlled LDLc [5]. A sub-analysis of the SPARCL (Stroke Prevention by Aggressive Reduction in Cholesterol levels) trial showed that, in the group randomized to atorvastatin, elevated lipoprotein(a) concentrations were positively and independently associated with an increased risk of coronary events (HR: 1.607 (95 % CI: 1.007–2.563) [6]. In post–acute coronary syndrome (ACS) patients enrolled in ODYSSEY Outcomes (Evaluation of Cardiovascular Outcomes After an Acute Coronary Syndrome During Treatment With Alirocumab), a 1 mg/dL reduction in lipoprotein(a) mass was associated with a HR of 0.994 (95 % CI: 0.990–0.999) for ASCVD events [7].

**Fig. 1:**
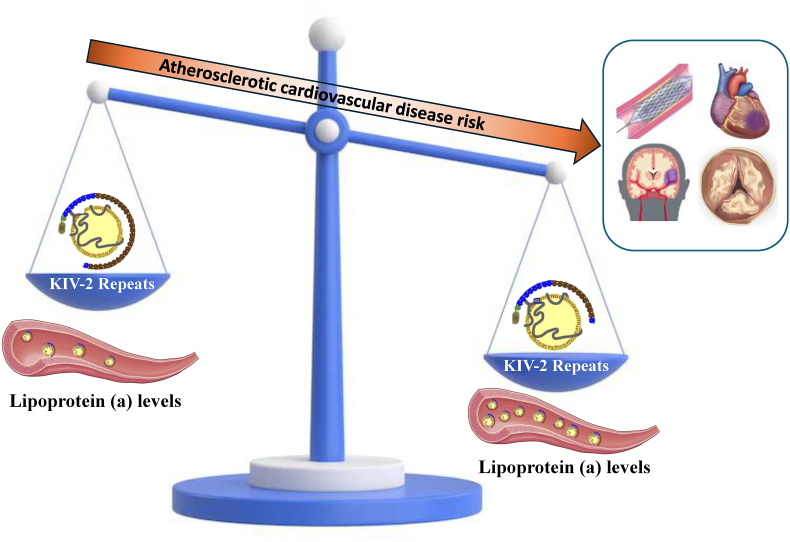
Schematic representation of the relationship between apolipoprotein(a) kringle IV type 2 repeats and lipoprotein(a) levels, and between these levels and atherosclerotic cardiovascular risk.

However, while large-scale cohort studies consistently show increased risk related to elevated lipoprotein(a) (Fig. 1), there has been limited validation of these findings in the clinical setting, or evaluation of risk thresholds that could be appropriate for targeting therapies in future clinical trials and practice. The presence of ASCVD and severe hypercholesterolaemia may identify patients who are more likely to be detected with elevated lipoprotein(a) when tested [8]. In this scenario, less than 0.5 % of people undergo lipoprotein(a) testing [9], a testing rate that is considered low especially among primary prevention patients with borderline-to-intermediate ASCVD risk [10].

Therefore, the aim of this review is to summarize the most recent evidence supporting the causal role of lipoprotein(a) in ASCVD and to explore potential treatments that can reduce the lipoprotein(a) burden until the first clinical trial outcomes become available [11].

## 2 An overview of the structure and circulating levels of lipoprotein(a)

Lipoprotein(a) is a complex lipoprotein consisting of a low-density lipoprotein (LDL)-like particle, which includes one molecule of apolipoprotein (apo)B-100, covalently linked to apo(a), a glycoprotein distinguished by its repeated “kringle” structures (reminiscent of a Scandinavian pastry). Kringles are present in numerous proteins that play a role in the coagulation and fibrinolytic pathways, and they have the ability to attach to fibrin in blood clots [12]. Apo(a) includes 10 different KIV subtypes (designated from 1 to 10) with high interdomain homology, a KV domain, and a serine protease domain. Nine of the 10 KIV types (1, 3–10) are present in a single copy, while the number of KIV type 2s (KIV-2s) can vary between 1 and more than 40. Low molecular weight isoforms (22 or fewer total KIV repeats) are associated to 4- to 5-fold higher lipoprotein(a) plasma levels compared to high molecular weight isoforms (more than 22 KIV repeats), with low variability in concentration within families [13]. The varying number of KIV-2 repeats is associated with the molecular characteristics of *LPA*, the gene encoding apo(a), which covers a region of over 130 kb on chromosome 6 (160,531,483–160,666,375 in the current human genome reference sequence hg38). The KIV-2 domain is encoded by a copy number variation that can generate over 30 gene alleles, giving rise to multiple protein isoforms (approximately 200–800 kDa) within the population [14]. This variation in DNA fragment size reflects the size diversity of protein isoforms in plasma, and both are co-inherited within families [15].

### 2.1 Genetic determinants of lipoprotein(a) levels

Among the single nucleotide polymorphisms (SNPs) in the *LPA* gene that are associated to increased or decreased lipoprotein(a) levels, rs10455872 and rs3798220 have garnered significant attention for their association with low molecular weight isoforms. These SNPs are strongly correlated with elevated lipoprotein(a) levels and a higher risk of coronary disease [16]. A recent replication study involving 440,368 UK Biobank participants of European ancestry confirmed that carrying one minor allele led to an increase in median lipoprotein(a) levels from 14 nmol/L to 146 nmol/L, while carrying two minor alleles resulted in median levels of 262 nmol/L. Higher lipoprotein(a) levels correspond to higher hazard ratios (HR) for ASCVD, specifically 1.47 for one minor allele and 1.89 for two minor alleles [17]. Given the extensive research on genetic variants that influence lipoprotein(a) levels, including candidate genes studies, sequencing, and GWAS, readers are encouraged to refer to the thematic review written by Coassin & Kronenberg for more detailed information [18].

### 2.2 Non-genetic determinants of lipoprotein(a) levels

While lipoprotein(a) levels exhibit minimal variation between men and women and remain fairly stable over the course of life [19], they are 17 % higher in women than in men after the age of 50, which generally coincides with the onset of menopause [20]. Conversely, lipoprotein(a) levels do differ according to race and ethnicity (i.e. individuals of African descent have the highest lipoprotein(a) levels among all studied racial/ethnic groups, followed by South Asians, Caucasians, Hispanics and East Asians) [21].

Elevated lipoprotein(a) levels have been observed in patients with various inflammatory conditions, such as rheumatoid arthritis, systemic lupus erythematosus, Crohn's disease, acquired immunodeficiency syndrome, chronic renal failure, and pulmonary arterial hypertension [22]. In patients with acute myocardial infarction, lipoprotein(a) levels were observed to increase, more than doubling within the first days following the cardiovascular event, regardless of their initial lipoprotein(a) values [23]. The *LPA* gene contains a class II interleukin 6 (IL-6) response element (CTGGGA), which accounts for upregulation of apo(a) expression [24]. In patients with rheumatoid arthritis, prolonged treatment with tocilizumab lowered lipoprotein(a) levels by 30–40 % on average [25]. Conversely, inconsistent results were found in patients with SARS-CoV-2 infection [[26], [27], [28], [29]].

Patients with metabolic dysfunction-associated liver steatosis exhibited lower levels of lipoprotein(a), possibly due to an impaired lipoprotein assembly [30], while those undergoing haemodialysis displayed elevated levels, likely resulting from reduced clearance rather than increased lipoprotein(a) production [31].

Observational and epidemiological studies indicate an inverse relationship between plasma lipoprotein(a) levels and risk of diabetes. Generally, this relationship appears nonlinear across the full range of lipoprotein(a) levels, with a sharp increase in risk only at very low concentrations, levelling off at moderate and higher values [32]. Data from two-sample Mendelian randomization analysis involving 563,420 patients established a causal link between lipoprotein(a) and type 2 diabetes mellitus [33]. An additional Mendelian randomization analysis indicated that hyperinsulinemia, commonly associated with type 2 diabetes mellitus, could have been partially responsible for the inverse relationship between low lipoprotein(a) levels and the increased risk of type 2 diabetes mellitus [34]. However, elevated lipoprotein(a) levels can be an independent and incremental risk factor for ASCVD outcomes in both diabetic and non-diabetic individuals [35]. Indeed, data of the EASSURE-NIRS registry showed that lipoprotein(a) might be associated with more vulnerable coronary atheroma in patients with diabetes despite receiving statin therapy. Even in patients with diabetes with LDLc <70 mg/dL, lipoprotein(a) levels remained associated with maximum 4-mm lipid-core-burden index in target lesions [36].

Historically, it is noted that lipoprotein(a) levels rise in overt hypothyroidism and decrease rapidly with triiodothyronine supplementation in patients with hypothyroidism [37].

Lifestyle modifications are crucial for ASCVD risk management. Evidence from well-controlled clinical studies suggests that certain dietary habits, particularly reducing saturated fatty acid intake, influence lipoprotein(a) levels [38]. In the context of obesity, fasting (voluntary abstinence from some or all foods and beverages) [39] does not seem to affect lipoprotein(a) levels [40], while bariatric surgery does [41,42].

### 2.3 Challenges in lipoprotein(a) measurements

Although it is now known that individuals with small isoforms (up to 22 KIV-2 repeats) possess lipoprotein(a) concentrations which are 4–5 times higher than those with only large isoforms (more than 22 KIV-2 repeats) [15], standardizing biochemical quantification for clinical use remains challenging. None of the available commercial assays for lipoprotein(a) quantification are completely isoform-insensitive [43]. This further contributes to the unsolved issue of how to express circulating levels of lipoprotein(a). Since all lipoproteins consist of lipids and proteins, one can refer to any single chemical species (e.g., cholesterol, triglyceride, protein), several, or all components combined using the term ‘mass’. Lipoprotein(a) is primarily composed of two proteins: apoB (with a molecular mass of 530 kDa) and apo(a) (whose mass ranges from about 200 to 800 kDa depending on the number of KIV-2 repeats). The non-protein components of lipoprotein(a) include lipids (mainly free and esterified cholesterol, triglycerides, and phospholipids) and carbohydrates (estimated to be between 4 % and 10 % of apoB mass). Additionally, small amounts of fat-soluble vitamins are likely present within the LDL core of lipoprotein(a). These factors determine the true mass of the lipoprotein(a) particle for each individual, rendering conversions from lipoprotein(a) ‘mass’ measurements to molar (i.e., particle) concentration approximate and imprecise. Such conversions should be avoided in order to guarantee accuracy [44]. Until these technical issues are solved, companies should not be required to report in molar units unless their assays are suited for this purpose. Providing companies with easily accessible reference materials measured by molar-based methods [45] will be a crucial step for enhancing assay performance [46]. Meanwhile, the role of lipoprotein(a) measurement as a risk factor for major adverse cardiovascular events remains undisputed. This is supported by a sub-analysis of the ODYSSEY Outcome trial (Evaluation of Cardiovascular Outcomes After an Acute Coronary Syndrome During Treatment With Alirocumab), with alirocumab, which demonstrated the prognostic cardiovascular value of lipoprotein(a) regardless of the assay used, including Siemens N-latex nephelometric immunoassay (mass – mg/dL), Roche Tina-Quant turbidimetric immunoassay (molar – nmol/L), and mass spectrometry (nmol/L) [47].

## 3 Cardiovascular risk associated to lipoprotein(a) levels – last updates (from 2021 to 2024)

Traditionally, two lipoprotein(a) cut-offs, 30 mg/dL and 50 mg/dL, have been used to identify individuals at elevated cardiovascular risk. Levels below 30 mg/dL are considered low, between 30 and 50 mg/dL can be considered intermediate, and above 50 mg/dL are considered elevated. The 50 mg/dL threshold roughly corresponds the 80th percentile for the population. However, these thresholds should be taken into account up to a certain point, as Patel et al. have demonstrated how cardiovascular risk arises at much lower levels of lipoprotein(a), and increases linearly with higher levels [48]. In this context, despite average or median lipoprotein(a) levels vary globally and by ancestry, the relative risk associated with baseline lipoprotein(a) levels seems roughly similar among groups studied so far [49]. However, the burden of ASCVD related to elevated lipoprotein(a) levels may be more than twice as high among individuals of African descent compared to Caucasian individuals [50].

Although different cut-offs have been employed by different National Societies, there is alignment in recommending lipoprotein(a) measurement as a routine part of clinical assessment in high-risk individuals. There is now sufficient evidence to support the recommendation to measure lipoprotein(a) levels at least once in every adult for risk stratification [17,[51], [52], [53], [54]]. This was further confirmed by the extended follow-up analysis of the Women's Health Study (WHS), which indicated that lipoprotein(a) levels could predict the occurrence of initial major cardiovascular events over a period of 30 years (HR = 1.06, 95%CI 1.03–1.08) [55].

The impact of this paradigm shift is timely and crucial compared to earlier guidelines, which on the other hand limited the recommendation for lipoprotein(a) measurement only to individuals with intermediate or high risk. Measuring lipoprotein(a) helps identify patients who may later benefit from specific lipoprotein(a)-lowering therapies [56]. Since lipoprotein(a) levels are inherited in an autosomal dominant manner, screening first-degree relatives of patients with high lipoprotein(a) can identify more at-risk individuals who may need intervention [57]. The findings of a cross-sectional study involving 52,418 suggested that the yield of cascade screening of first-degree relatives of individuals with high lipoprotein(a) levels was over 40 %. The odds ratio (OR) of relatives having high lipoprotein(a) levels when their index relative also had high lipoprotein(a) levels, compared to those whose index relatives did not, was 7.4 (95%CI, 6.8–8.1) for first-degree relatives and 3.0 (95 % CI, 2.7–3.4) for second-degree relatives [58]. Conversely, lipoprotein(a) remains a modest contributor to cardiovascular polygenic risk [59].

Genome-wide association studies using the UK Biobank population have demonstrated that the atherogenicity of lipoprotein(a) is substantially greater than that of LDL. The strength of these studies resides in the fact that the association with cardiovascular events was assessed according to the apoB component. The risk was determined by examining the genetic variation in apoB within the lipoprotein(a) particle. This involved identifying single nucleotide polymorphisms (SNPs) linked to differences in lipoprotein(a) mass concentration, which had been biochemically measured, and then calculating the actual size (beta coefficients) of these specific SNPs in plasma apoB levels. The atherogenic potential of lipoprotein(a) was found to be about 6-fold higher than that of LDL on a per-particle basis (point estimate of 6.6; 95%CI: 5.1–8.8) [60]. In line with these findings, a retrospective registry analysis indicated that among 23,398 individuals with lipoprotein(a) levels above 90 mg/dL, the HR was 1.25 (95%CI 1.05–1.50) for major adverse cardiovascular events, 1.37 (95%CI 1.14–1.64) for ASCVD, and 1.62 (95%CI 1.28–2.05) for coronary artery disease, compared to individuals with lipoprotein(a) levels below 17 mg/dL [61]. Accordingly, a cross-sectional case-control study conducted at Amsterdam UMC, a tertiary hospital in The Netherlands, found that patients with lipoprotein(a) levels above 459.6 nmol/l had an OR of 3.4 for myocardial infarction and 2.6 for ASCVD compared to age- and sex-matched patients with lipoprotein(a) levels of 7 nmol/L [62]. This is a notable finding, given that having extremely high levels of lipoprotein(a) is about two to three times more common than the occurrence of heterozygous familial hypercholesterolemia (FH). Despite this knowledge, a multicentre cross-sectional epidemiological study involving 48,135 patients with a history of ASCVD found that lipoprotein(a) levels had been measured only in a small fraction of them (14 %) [63].

In relation to the link between lipoprotein(a) and systemic inflammation, two recent studies in primary and secondary prevention settings indicate that the inflammatory burden influences the prognostic ability of lipoprotein(a). They found that elevated lipoprotein(a) levels were associated with future ASCVD risk only in individuals with residual inflammatory risk (i.e. high-sensitive C-reactive protein (hsCRP) levels >2 mg/L) [64,65]. Specifically, a sub-analysis of the ACCELERATE study (Assessment of Clinical Effects of Cholesteryl Ester Transfer Protein Inhibition With Evacetrapib in Patients at a High Risk for Vascular Outcomes) revealed that each unit increase in log of lipoprotein(a) levels was associated with a 13 % higher risk of cardiovascular death, nonfatal myocardial infarction, or stroke only in those with hsCRP levels ≥2 mg/L^64^. The MESA study (Multi-Ethnic Study of Atherosclerosis) demonstrated that in primary prevention, significant ASCVD risk was observed in the case of levels lipoprotein(a) of 50–99.9 mg/dL (HR: 1.36; 95%CI: 1.02–1.81) and ≥100 mg/dL (HR: 2.09; 95%CI: 1.40–3.13), but again, only in individuals with hsCRP ≥2 mg/L [65]. However, these findings were either based on post-hoc analysis in a highly selected study population with established ASCVD [64] or conducted within a multi-ethnic population with significant variation in lipoprotein(a) levels and relatively low number of cardiovascular events [65]. Conversely, data from a study performed on 68,090 individuals of the Danish general population showed that high lipoprotein(a) was a major driver for the risk of ASCVD, myocardial infarction, and aortic valve stenosis at both high and low CRP levels [66].

To clarify this issue, Arnold et al. conducted an analysis of individuals without (65,661) and with (6017) established ASCVD at baseline, following them for 9.8 and 13.8 years, respectively. Among ASCVD-free individuals, lipoprotein(a) was significantly linked to new ASCVD cases regardless hsCRP levels. However, in participants with ASCVD at baseline, lipoprotein(a) was associated with recurrent ASCVD events only in those with residual inflammatory risk (hsCRP ≥2 mg/L; HR = 1.34 (95%CI 1.03–1.76)) [67].

While awaiting the results of the cardiovascular outcome trials [68,69], the specific patient population most affected by elevated levels and who might benefit from targeted therapeutic interventions remains undefined. Additionally, the threshold of elevated lipoprotein(a) that would be most effective for identifying higher-risk cohorts for targeted intervention in primary prevention is still unclear. In this context, Berman et al. examined the relationship between lipoprotein(a) and major adverse cardiovascular events in patients with and without baseline ASCVD. For individuals with a history of ASCVD, there is a significant increase in incident ASCVD, simultaneously associated with higher lipoprotein(a) levels, and the highest risk was observed when lipoprotein(a) exceeded 53 mg/dL, a threshold lower than that used in current trials [70]. For those without a history of ASCVD, a higher lipoprotein(a) threshold (i.e., >100 mg/dL) was needed to identify those at elevated risk [71]. The analysis of five major U.S. prospective primary prevention cohorts further highlighted the predictive value of lipoprotein(a) for future ASCVD events, which, although similar in individuals at low to intermediate risk compared to higher-risk primary prevention groups, is greater in patients with diabetes mellitus [72]. Considering lipoprotein(a) levels of 3.6 mg/dL as the reference value of 13,835 individuals, the HR for ASCVD was 1.06 (95%CI 0.99–1.14) for lipoprotein(a) levels of 13.5 mg/dL, 1.18 (95%CI 1.09–1.28) for levels of 25.9 mg/dL and 1.46 (95%CI 1.33–1.59) for levels of 52.6 mg/dL [72].

In the context of lipoprotein(a)-driven cardiovascular risk, another unsolved issue is the importance of adjusting LDLc values for the cholesterol carried by lipoprotein(a) (i.e., LDLc without considering its lipoprotein(a)-C content). Typically, 30 % of lipoprotein(a)-C composition has been estimated to be made up of lipoprotein(a) mass, although this correction has been questioned by Yeang et al. [73]. An analysis of 68,748 ASCVD-free subjects at baseline concluded that adjusting LDLc for its lipoprotein(a)-C content did not significantly alter LDLc–associated cardiovascular risk estimation within the population [74]. However, as noted in the accompanied editorial, the average lipoprotein(a) value was only 9.3 mg/dL, and the top decile of lipoprotein(a) levels was only 43.5 mg/dL, significantly lower than what is considered elevated ASCVD risk and less than half of what has been observed in other European populations. Thus, empirically measuring lipoprotein(a)-C and adjusting for this fraction in LDLc is necessary to define the contribution of corrected LDLc in observational studies and clinical trials across a broad spectrum of populations [75].

In evaluating atherosclerotic burden, a validated measurement which guides primary ASCVD decision is the coronary calcium score (CAC). Data from the MESA study indicated that lipoprotein(a) and CAC were independently associated with ASCVD risk and could be useful for guiding primary prevention therapy decisions. Compared to participants with non-elevated lipoprotein(a) and CAC = 0, those with elevated lipoprotein(a) and CAC >100 were at the highest risk (HR: 4.71; 95%CI: 3.01–7.40), and those with elevated lipoprotein(a) and CAC = 0 were at a similar risk (HR: 1.31; 95%CI: 0.73–2.35) [76]. In patients with advanced stable coronary artery disease undergoing coronary computed tomography angiography at baseline and 12 months to assess progression of total, calcific, noncalcific, and low-attenuation plaque, lipoprotein(a) > 70 mg/dL was associated with accelerated progression of the necrotic core [77].

However, the extent of lipoprotein(a) reduction required to achieve a clinically significant benefit is unknown, and therapies that lower lipoprotein(a) levels and reduce cardiovascular events in a targeted manner are still under investigation. Genetic association and Mendelian randomization using *LPA* variants as genetic instruments have suggested that a substantial absolute decrease in lipoprotein(a), from 65 to 100 mg/dL, equivalent to a 1-mmol/L decrease in LDLc, would be necessary to achieve the clinical benefits on cardiovascular outcomes.

An interesting case involves patients with FH [78]. Epidemiological studies have shown that about one-third of FH patients have elevated levels of lipoprotein(a), defined as >50 mg/dL, and around 5 % have levels above 100 mg/dL, compared to 2.6 % in non-FH hypercholesterolemic patients. Elevated lipoprotein(a) in FH creates a unique situation where two genetically determined proatherogenic factors contribute to lifelong atherosclerotic cardiovascular risk burden [79]. Based on these findings, testing for elevated lipoprotein(a) levels during FH cascade screening helps identify relatives with high lipoprotein(a) and increased risk of ASCVD, especially when the proband has both FH and elevated lipoprotein(a) [80]. In line with this evidence, approximately one-quarter of those meeting the clinical diagnosis of FH in the Danish general population had high plasma concentrations of lipoprotein(a) [81]. Nonetheless, it must be kept in mind that FH does not directly cause elevated lipoprotein(a) levels; rather, elevated lipoprotein(a) increases the likelihood of clinical recognition of genetic FH [81]. Notably, findings of Ellis et al. demonstrated that testing for elevated lipoprotein(a) during cascade screening for FH was effective in identifying relatives with high lipoprotein(a) and increased risk of ASCVD, particularly in the case of probands with both FH and elevated lipoprotein(a) [82]. Among 162 relatives tested (136 adults and 26 children), the prevalence of FH and elevated lipoprotein(a) was 60.5 % and 41.4 %, respectively. Cascade testing identified a new case of FH, elevated lipoprotein(a) and FH with elevated lipoprotein(a) for every 1.5, 2.1 and 3.0 relatives tested, respectively [83]. Therefore, cascade testing from FH adult index cases with elevated lipoprotein(a) in a routine clinical setting is a feasible and effective approach for identifying new cases of FH and detecting new cases of elevated lipoprotein(a) among children and adolescents [84].

## 4 A brief overview of the most recent direct approaches to lower lipoprotein(a) levels

Recent phase 1 and 2 clinical trials have shown promising results, and ongoing phase 3 trials are working on the introduction to the market of specific lipoprotein(a)-lowering drugs to reduce cardiovascular disease risk [85]. Clinical trials have demonstrated efficacy of RNA-interfering therapies that reduce apo(a) synthesis, and of small molecule inhibitors that disrupt the bonding of apo(a) to apoB [70,86,87]. Since this topic is beyond the scope of the present review, readers are encouraged to refer to literature specifically focused on lipoprotein(a)-lowering drugs [86,88,89].

Briefly, the molecules being tested in preclinical and clinical studies include pelacarsen (antisense oligonucleotide), olpasiran (short interfering RNA), zerlasiran (short interfering RNA), lepodisiran (short interfering RNA), muvalaplin (small molecule inhibitor) and CTX320 (a gene-editing approach using lipid nanoparticle delivery of Cas9 mRNA and gRNA to the liver) (Table 1). Additionally, upcoming phase 3 trials testing the hypothesis that lowering lipoprotein(a) reduces cardiovascular diseases include the Lp(a) HORIZON with pelacarsen (NCT04023552), OCEAN(a) with olpasiran (NCT05581303), and ACCLAIMLp(a) with lepodisaran (NCT06292013) [90] (Table 2). However, as noted elsewhere, if any of these trials result in beneficial outcomes, long-term safety data will be required in patients achieving ultralow (<5 mg/dL) or unmeasurable lipoprotein(a) levels with pharmacological therapies, given that the physiological function of lipoprotein(a) is unknown [90].

**Table 1:** Current strategies for directly lowering lipoprotein(a).

Drug	Mechanism	Efficacy
Pelacarsen	It is a 2′-Methoxyethyl gapmer chimeric antisense oligonucleotide (ASO) targeting LPA mRNA. It binds to the exon 24:25 splice site of the mature human Apo(a) mRNA, at position 3901–3920 with the unique sequence (CTTGTTCTGCTCCGTTGGTG) that is absent in the mRNA of plasminogen. This site also lacked any known single nucleotide polymorphisms that might limit efficacy in specific individuals with these genetic variations [108]. The N-acetyl-galactosamine (GalNAc) complex is covalently attached with a proprietary linker to the 5′ end, allowing for rapid and specific uptake within hepatocytes via the asialoglycoprotein receptor.	Lipoprotein(a) levels are reduced up to 80 % [70]
Olpasiran	It is a tri-antennary N-acetyl-galactosamine (GalNAc)-conjugated short interfering RNA (siRNA) directed against the mRNA of the LPA gene. Olpasiran is modified with 2′-fluoro and 2′-methoxy substitutions and phosphorothioate internucleotide linkages at the termini to stabilize the duplex [109].	Lipoprotein(a) levels are reduced more than 95 % [86]. Its pharmacodynamic effects are reversible and persistent effects on lipoprotein(a) lowering continued to be seen through 1 year after discontinuation of olpasiran [110].
Zerlasiran (SLN360)	It is a siRNA targeting LPA messenger RNA. SLN360 is a 19-mer siRNA covalently linked to a tri-antennary N-acetyl-galactosamine (GalNAc) moiety [111].	Lipoprotein(a) levels are reduced up to 98 % [111].
Lepodisiran (LY3819469)	GalNAc-conjugated mixed 2′-O-Me, 2′-fluoro and unmodified Dicer siRNA (DsiRNA; a sense strand with tetraloop hairpin, a constant sequence, and a 21–23 nt antisense strand complementing the LPA target mRNA) [112]	Lipoprotein(a) levels are reduced more than 95 % [113]
Muvalaplin (LY3473329)	It is the first-in-class small molecule inhibitor of lipoprotein(a) formation, disrupting the initial non-covalent interaction between apo(a) Kringle IV domains 7 and 8 and apoB. The ability to disrupt this interaction mimics naturally occurring genetic apo(a) variants that prevent interaction with apoB and result in low lipoprotein(a) levels [87].	Lipoprotein(a) levels are reduced up to 65 % following daily administration for 14 days [114]
CTX320	Cas9 mRNA and guide RNA encapsulated within a lipid nanoparticle	A single infusion of 2 mg/kg led to significant and durable decreases in plasma lipoprotein(a) levels up to 94 % [115].

**Table 2:** Ongoing phase 3 trials evaluating the outcomes of lipoprotein(a)-lowering drugs.

Drug	Trial acronym	Population characteristic	Primary endpoint
Pelacarsen	Lp(a) HORIZON (NCT04023552)	1) Enrolment: 8323 2) ASCVD patients (myocardial infarction or ischemic stroke 3 months to 10 years from screening; clinically significant symptomatic peripheral artery disease) 3) Lipoprotein(a) ≥70 mg/dL at the screening visit	Time to first occurrence of cardiovascular death, non-fatal myocardial infarction, non-fatal stroke and urgent coronary re-vascularization requiring hospitalization in patients with lipoprotein(a) ≥ 70 mg/dL and ≥90 mg/dL.
Olpasiran	OCEAN(a) (NCT05581303)	1) Enrolment: 7297 2) History of ASCVD 3) Lipoprotein(a) levels ≥200 nmol/L (more than ≈80 mg/dL)	Time to coronary heart disease death, myocardial infarction, or urgent coronary revascularization, whichever occurs first.
Lepodisaran	ACCLAIMLp(a) (NCT06292013)	1) Estimated enrolment: 12500 2) a) Patients with established ASCVD with an event or revascularization b) Individuals 55 years of age or older with documented coronary artery disease, carotid stenosis, or peripheral artery disease without history of event or revascularization; known familial hypercholesteremia; or a combination of high-risk factors. 3) Lipoprotein(a) levels ≥175 nmol/L	Time to First Occurrence of Any Component of the MACE-4 Composite Endpoint (cardiovascular death, nonfatal myocardial infarction, nonfatal stroke, and urgent coronary revascularization).

HORIZON, Assessing the impact of lipoprotein (a) lowering with pelacarsen (TQJ230) on major cardiovascular events in patients with CVD; OCEAN(a), Olpasiran Trials of Cardiovascular Events and Lipoprotein(a) Reduction; ACCLAIMLp(a), A Study to Investigate the Effect of Lepodisiran on the Reduction of Major Adverse Cardiovascular Events in Adults With Elevated Lipoprotein(a).

ASCVD, atherosclerotic cardiovascular diseases; MACE, Mayor Adverse Cardiac Events.

Although not in the remit of the present paragraph, besides statins, which are known to slightly increase lipoprotein(a) levels [91], it is important to highlight the effect of the cholesteryl ester transfer protein (CETP) inhibitor, obicetrapib, on lipoprotein(a) levels [92]. The ROSE study (Randomized Study of Obicetrapib as an Adjunct to Statin Therapy) demonstrated that an 8-week treatment with obicetrapib at doses of 5 mg and 10 mg reduced lipoprotein(a) levels by 33.8 % and 56.5 %, respectively, in patients with LDL-C >70 mg/dL and triglycerides <400 mg/dL, who were following a stable, high-intensity statin regimen [93]. This finding was corroborated by the ROSE2 trial (Study to Evaluate the Effect of Obicetrapib in Combination with Ezetimibe as an Adjunct to High Intensity Statin Therapy), in which it was observed that a 10 mg dose of obicetrapib resulted in a 47.2 % reduction in lipoprotein(a) levels from baseline [94].

Finally, although lipoprotein apheresis is a particular treatment for patients with difficulty managing dyslipidaemia, it remains the only FDA-approved option for those with elevated lipoprotein(a) and progressive ASCVD who do not respond to optimal medical therapy. This treatment can reduce lipoprotein(a) levels by more than >50 % [95,96]. For patients with refractory angina and elevated lipoprotein(a) levels, lipoprotein apheresis has resulted in improvements in myocardial perfusion, atheroma burden, exercise capacity and angina symptoms [97].

## 5 Concluding remarks

With over 1.4 billion people worldwide having elevated lipoprotein(a) levels, and with knowledge of the associated risks, identifying those more likely to have higher levels is crucial [98]. In this context, efforts are needed to overcome the common barriers to ordering the lipoprotein(a) test, such as lack of reimbursement, limited treatment options, and high laboratory costs [99]. Thus, utilizing new strategies (e.g., artificial intelligence) can potentially increase the effectiveness of lipoprotein(a) testing by over twofold, with a more significant improvement among those without ASCVD compared to those with ASCVD. The ARISE model (Algorithmic Risk Inspection for Screening Elevated lipoprotein(a)), based on data from the UK Biobank (N = 456,815), and from 3 large cohort studies, ARIC (N = 14,484), CARDIA (N = 4124) and MESA (N = 4672), demonstrated a reduction in the number needed to test to find one individual with elevated lipoprotein(a) by up to 67.3 % [100]. In this context, it is essential to recognize the ongoing challenges associated with implementing a lipoprotein(a) familial cascade screening strategy in clinical practice. Key steps include establishing threshold values for initiating cascade screening, accounting for ethnic variability in lipoprotein(a) levels, and conducting further research into the clinical significance of cascade screening [101].

Without specific targeted treatments to lower lipoprotein(a), recommendations for individuals with elevated lipoprotein(a) focus on overall cardiovascular risk reduction. More intense LDLc lowering and control of dyslipidaemia and other ASCVD risk factors are suggested for patients with elevated lipoprotein(a) [4]. It is advised to prescribe lipoprotein(a) measurements (i) in all adults at least once in life, (ii) in young individuals with a history of ischaemic stroke or a family history of premature ASCVD or high lipoprotein(a) with no other identifiable risk factors, (iii) during cascade testing for high lipoprotein(a) in the context of FH, and (iv) in patients with calcific aortic valve stenosis [102]. This approach is crucial, as ignoring a potent risk factor like high lipoprotein(a) levels will inevitably lead to major misclassifications of cardiovascular risk within the population [62]. Accurate measurement of lipoprotein(a) is also important for establishing a threshold high enough to be associated with clinical risk and for intensifying preventive therapies [103].

According to an analysis reported in the 2022 European Atherosclerosis Society consensus paper, if a person has a baseline estimated lifetime risk of 10 % and a lipoprotein(a) concentration of 75 mg/dL, the risk increases by an additional 6.5 %–16.5 % compared to someone with lipoprotein(a) concentration below the median of 7 mg/dL [17]. Therefore, until the implementation of therapies capable of lowering lipoprotein(a) to levels required for cardiovascular benefit, it is recommended to promote and reinforce healthy metrics (Life's Essential 8), while preventing decline in those with potential for unfavourable trajectories [104]. Within the context of these ideal cardiovascular health metrics, data of the EPIC-Norfolk Study demonstrated that among participants with lipoprotein(a) concentrations above 50 mg/dL, those with a low number of concomitant risk factors had only one third of cardiovascular risk over the subsequent 11.5 years compared to those with a high number of risk factors [105]. Specifically, among patients with lipoprotein(a) > 50 mg/dL, those with ideal cardiovascular health have a relative risk of ASCVD of 0.33 (95%CI, 0.17–0.63) compared to those with poor cardiovascular health [105]. Furthermore, it is crucial to acknowledge the role of lipoprotein(a) in secondary prevention. Individuals with elevated lipoprotein(a) also face a higher risk of adverse outcomes following percutaneous coronary intervention (PCI) for in-stent restenosis (ISR). In a study involving over 1200 patients with ISR, those with lipoprotein(a) above 30 mg/dL were linked to cardiovascular events over a median follow-up of 3 years [106]. Supporting this observation, a retrospective study found that among 1209 patients who underwent successful PCI, elevated lipoprotein(a) levels were independently associated with ISR events (HR = 1.67, 95 %CI 1.18–2.37), with this association becoming more pronounced after the first year post-PCI [107].

## Funding

This work was partially supported by Ministero dell'Università e della Ricerca (Progetti di Rilevante Interesse Nazionale) 2022ZW3M8H (to A.C.) and 2022ZPS49L (to M.R. and S.C.).

## Declaration of competing interest

The authors declare that they have no known competing financial interests or personal relationships that could have appeared to influence the work reported in this paper.
